# “It’s a revolving door”: understanding the social determinants of mental health as experienced by formerly incarcerated people

**DOI:** 10.1186/s40352-023-00227-8

**Published:** 2023-06-10

**Authors:** Shivani Nishar, Esteem Brumfield, Shromona Mandal, Rahul Vanjani, Jon Soske

**Affiliations:** 1 Center for Health and Justice Transformation, Rhode Island Hospital, Providence, RI USA; 2 School of Public Health, Brown University, Providence, RI USA; 3 Department of American Studies, Brown University, Providence, RI USA; 4 Division of General Internal Medicine, Warren Alpert Medical School of Brown University, Providence, RI USA; 5 Division of Addiction Medicine, Lifespan; Center of Biomedical Research Excellence (COBRE) on Opioids and Overdoses, Miriam Hospital, Providence, RI USA

**Keywords:** Re-entry, Mental health, Incarceration, Social determinants of health, Qualitative research

## Abstract

**Background:**

This qualitative study seeks to understand how formerly incarcerated individuals in Rhode Island conceptualize their mental health and perceive obstacles to accessing and utilizing mental health services following recent incarceration.

**Methods:**

We conducted in-depth semi-structured interviews from 2021 to 2022 with 25 people who had been released from incarceration within the past five years. We identified participants using voluntary response and purposive sampling. We analyzed the data using a modified form of grounded theory developed to capitalize on insights drawn from the lived experience of research team members, including a team member with experience of incarceration, and refined initial findings with a community advisory board with lived experience of incarceration and/or mental health issues similar to the study’s sample.

**Results:**

Participants overwhelmingly identified social determinants of health such as housing, employment, transport, and insurance coverage as the main obstacle to both accessing and maintaining engagement with mental health care. They also reported a level of opacity in the mental health system as they attempted to navigate it with limited systems literacy and support. Participants discussed alternative strategies that they employed when they believed formal mental health failed to meet their needs. Importantly, the majority of participants perceived a lack of empathy or understanding from their providers regarding the impact of SDOH on their mental health.

**Conclusions:**

Despite growing efforts to address social determinants among formerly incarcerated people, the majority of participants believed that providers neither understood nor addressed these dimensions of their lives. Participants reported two social determinants of mental health that have not yet been adequately explored in the literature: mental health systems literacy and systems opacity. We offer some strategies for how behavioral health professionals can develop stronger relationships with this population.

## Introduction

There is a growing importance placed on understanding the mental health needs of people who are incarcerated, with academics, criminal legal system reformers, and clinicians alike advocating for robust mental health services within prisons and jails. A wealth of evidence emphasizes the need for such concern. In 2011–2012, the U.S. Department of Justice reported that 44% of people in jail and 37% of people in state and federal prisons had a history of mental illness’(Bronson & Berzofsky, 2017).

The fragmented federal, state, and local/county carceral system renders it impossible to reliably collect overall statistics about the impact of incarceration on mental health, though there is evidence that concretely shows different forms of incarceration having a negative effect. People with mental illness and/or substance use disorder suffer disproportionate rates of incarceration, with an estimated 2 million people with mental illness and substance use disorder being booked into jails every year (NAMI, 2021), and ample literature explains how the conditions of confinement itself further exacerbates mental illness. Solitary confinement, for example, which is used in at least 44 states, including Rhode Island (American Civil Liberties Union, 2014), can result in symptoms including hallucinations, paranoia, psychosis, and violent fantasies (Smith, 2006). Incarcerated individuals also experience and witness violence within their living environment, creating acute trauma with potential for post-traumatic stress disorder. On average, 35% of men and 24% of women experience physical violence while 10% of men and 25% of women are sexually harassed or assaulted in prison (Wolff et al., 2009). Incarcerated people are also forced to manage the compounding impacts of little autonomy (Vanjani, 2017), poor nutrition (Sarris et al., 2015) and hygiene, overcrowding, limited medical care, restricted social relationships (Chuang et al., 2013), and strict prison rules that punish individuals with mental illness (Fellner, 2006), all of which worsens mental health.

Mental health care remains largely inaccessible for people with mental health and/or substance use conditions who have been released from prison (Pew Center on the States, 2011). Those who are able to access care post-release face unique, additional barriers to maintaining engagement with treatment overtime. Studies have shown that both people with severe mental illness (Knaak et al., 2017) and a criminal record (Frank et al., 2014) are more likely to be discriminated against in mental health care settings. The institutional setting of most mental health services can itself act as an obstacle: patients with incarceration histories have described hospitals as having a prison-like environment, negatively impacting their care (Rotter et al., 2005, Simon et al., 2020). Formerly incarcerated people are also often systematically prohibited from employment opportunities and quality housing, both of which are substantiated by research as key social determinants of maintaining positive mental health and engaging with mental health services (Evans et al., 2003, Llosa et al., 2018).

While there is existing research on challenges accessing mental health care in prisons and jails, there is little research on obstacles to accessing mental health services post-release. Additionally, there has been a gap in exploring how formerly incarcerated people themselves view and experience barriers to accessing and engaging with mental healthcare mental health care. This study seeks to understand these experiences as conveyed by the participants themselves and on their terms. This is crucial since their perceptions informs the strategies that they utilize to access and navigate mental health care and, importantly, how they understand mental health care as failing them.

## Methods

Inclusion criteria consisted of individuals 18 years and older who had been released from incarceration within the past five years and self-identified as interfacing with mental health care in Rhode Island post-release. Each interview lasted 45–90 min and took place in-person at the Center for Health and Justice Transformation’s conference room in Providence, Rhode Island. All participants were asked open-ended questions about their experiences navigating mental health care post-release. Only the participant and the interviewer were present in the interview room. The interviewer had extensive training in mental health crisis response and trauma-informed care. Participants were paid thirty dollars in cash at the end of their interview. Verbal consent was obtained from each participant and each interview was audio recorded and transcribed verbatim.

The study broadly follows the “Consolidated criteria for reporting qualitative research (COREQ): a 32-item checklist for interviews and focus groups” to ensure comprehensive reporting (Tong et al., 2007).

### Data analysis

The transcripts were coded using a modified grounded theory approach; each identified theme and subtopic was assigned a code, allowing for a more rigorous and comparative interpretation of the interview data. Open and selective coding was used in order to understand the relationship between codes. The process of research was iterative, using emergent themes from past interviews to update future interviews. Interviews were independently coded using the constant comparative method by three researchers (S.N., E.B., S.M.) and then reconciled through discussion until intercoder agreement was achieved. This resulted in relative thematic saturation. A fourth team member (J.S.) met with the coding team after the completion of each batch of five interviews to discuss coding methodology, emerging themes, and theoretical questions raised by the data. Since we were concerned with how participants understood and reflected on their own experiences, and sought to center silenced or marginalized perspectives, analysis of data was further guided by critical phenomenology (Bronson & Berzofsky, 2017, Weiss et al., 2020).

Our team conducted this study using a community participatory research model and collaborated with community stakeholders. The team refined initial findings with Koren Carbuccia, a research advisor with lived experience with incarceration, and the Center of Biomedical Research Excellence (COBRE) on Opioids and Overdose Community Advisory Board, which is composed of peers with lived experience of mental illness, substance use, and incarceration.

### Demographics

Our participants included 25 people, 16 men and 9 women, who had been released from incarceration within the past 5 years and had identified themselves as having some interaction with mental health care (as defined by the participants themselves, including substance use disorder treatment) in Rhode Island post-release. Participants were over the age of 18 and able to speak English. We partnered with local advocacy groups, peer-led harm reduction organizations, recovery community centers, and reentry organizations to recruit eligible participants, including but not limited to Amos House, Crossroads, Direct Action for Rights and Equality, Formerly Incarcerated Union, House of Hope, Oasis Wellness and Recovery Center, Open Doors, Project Weber/RENEW, and Reentry Campus Program. Participants directly contacted the interviewer (S.N.) over the telephone, stating their interest in the study.

Forty-four percent of our study’s participants self-identified as white, 32% as Black, 12% as Hispanic, 8% as more than one race/ethnicity, and 4% as American Indian. At the time of the interviews, 36% resided in a recovery house, 28% were renting an apartment with one or two other people and 20% reported being currently homeless. Sixty percent of total participants had experienced homelessness at some point after their release. Participants self-reported having one or more diagnoses, with the top five being post-traumatic stress disorder (56%), anxiety (56%), depression (48%), substance use disorder (44%), and bipolar disorder (16%).

Participants described receiving services from a range of mental health settings, including but not limited to: community mental health centers, primary health clinics, Alcoholics and Narcotics Anonymous, recovery houses, addiction treatment centers, inpatient psychiatric units, outpatient psychiatric programs, private practices, housing services organizations, the VA and peer support groups.

## Results

### The overwhelming demands of life post-release

For many participants, being released from prison invoked a new set of stressors as they attempted to return home with minimal support infrastructure. One participant said that since being released, “I've been going through a lot of stuff over the past four years. I was homeless for a little while and had a lot of custody issues with my son. I mean I’ve just been going through a lot of stuff and I put all my health, all my medical on the backburner.” The large number of life issues that require attention post-release, sometimes with little support, made prioritizing and locating mental health care seem daunting. Participants described being overwhelmed by juggling medical appointments, searching for employment and housing, and meeting parole requirements, often feeling obligated to prioritize these other responsibilities over obtaining mental health care.

### Fragmentation of services

The fact that participants found services divided across agencies, with mental health agencies not equipped to address needs beyond therapy and medication, could compound both the stress of juggling post-release responsibilities and create the perception that agencies were not equipped to help people such as themselves. “There’s more happening with me and I need the help other than counseling. I did a lot of time in jail so I’m trying to find other areas that can deal with me, help me cope with my mental health,” a participant explained. “Like I’m on SSI, I’m waiting for that to get turned back on. And with that right there, they could probably help me get some sort of subsidized housing. Cause all that has to do with mental health, you know? So I’m trying to figure out what I can get from them. What types of mental health are they talking about? Is it just sitting down, talking to a therapist? Or is it more involved?" Several participants who sought out care at community mental health centers in an effort to be connected to case management services reported being ultimately disappointed with the poor quality of services they received. Provider understanding of mental health treatment was often narrower than how participants understood the issues involved in their mental health struggles; this led to the perception that providers did not fully understand, or in some cases did not want to help them address, the full range of challenges they faced post-incarceration.

### The social determinants of health that impact access and engagement with care

The majority of participants reported being homeless at one point after being released from incarceration. The role of housing instability in how participants articulated the obstacles to accessing services was a prominent theme. As one participant detailed: “It’s very hard when you’re on the street like that just to be able to survive, let alone take care of your mental health. When you’re in survival mode, you’re worried about where your next meal is gonna come from, where you’re going to sleep. You’re kind of always watching over your shoulder and it can be tough…it’s just like you don’t really have time for mental health.”

Participants who were homeless at the time of their interviews expressed frustration that either therapy and medications were offered as “solutions” to their housing insecurity or their provider seemed unable to incorporate the realities of their housing insecurity into the care provided. One participant reflected on continuing to go to her therapy appointments while homeless despite its ineffectiveness, saying, “She’s [therapist] really not very helpful with anything. I’ve been homeless for like years with my boyfriend and um we actually lived in a tent right across the street almost from the office cause I have to go there frequently. And everybody there knew it and she’s just oh, you know, how’s this going, how’s that going. And um because it’s really awkward talking to her, I’m like everything’s great! And clearly everything is not great.” While striking, it is also important to note that this anecdote was far from unique: several participants went to extraordinary lengths to engage mental health services despite both formidable obstacles and frustrations with the services that they received.

When thinking about a positive experience with mental health support, the same participant shared that she prefers to “unload” on her caseworker from House of Hope, a non-profit that supports homeless Rhode Islanders. “She knows where I’m coming from,” the participant continued. “She comes to my tent or where I’m sleeping outside you know? And brings us things that we need…I guess she kind of sees more…it’s a lot easier to say something and have her understand like what I mean, not just what I’m saying.”

### SDOH: employment

Being formerly incarcerated limited the housing options for several participants who attempted to rent an apartment post-release. A common experience was to spend years on waiting lists for subsidized housing units, recovery houses, and other residential programs. For the small number who managed to find housing through this route, finding employment in order to afford rent proved to be a precarious stopgap between housing and homelessness. “I’ve been going to job fairs but my [criminal] background is keeping me from jobs. I’ve lost jobs because of it in the last three months,” a participant said. “That’s what I’m getting the panic attacks about because I’ve got rent to pay…I’m trying to do everything I can.” Finding employment was also key to establishing the overall life stability needed to consistently engage with mental health care.

Without secure employment, most participants relied on government assistance or other means to financially provide for themselves. “I have no income. Just food stamps for the last 20–30 years, that’s it,” one participant said. Though she initially engaged in mental health care for her substance use, she elaborated how drug use allowed her to be in the state of mind she needed to engage in financially lucrative acts. “Been surviving all these years with just food stamps and the tools, I can say, that I learned through getting high. Hustling by any means necessary. Stealing. Prostitution.” At the same time, she shared that using substances led her to miss her counselling appointments, ultimately causing her to be kicked out of her program that had a policy of discontinuing care after three missed appointments. “They said that they cannot give me any services until next year.” These types of “double binds”—using substances to help achieve the financial stability needed to engage with care which also undermined engagement with care—were common throughout our interviews.

### SDOH: insurance

While incarcerated, no participants had insurance and several reported that they were independently responsible for enrolling in coverage once they were released. In the process of applying, waiting for, and then physically obtaining their insurance card, a few participants shared they went weeks without filling their prescriptions. “I'm waiting for that [insurance card] to come in the mail. It goes to a PO box and my people aren't around to open the PO box for me. I really can't see too many people without that insurance card or pick up a prescription,” a participant described. Another shared how her lack of income coupled with gaps in Medicaid coverage prevented access to lifesaving mental health care: “I went to go pick them [medications] up, I couldn't get them. Eighty something bucks, where am I getting $80? I don't got no income, I'm homeless, and my insurance didn't cover it, that's why they didn't give them to me. Like I need those, I could die." Limited insurance coverage impacted several participants; one participant shared that she wanted to remain at the in-patient program she had been enrolled in for four days but that she had to leave: “They don’t kick us out, they just tell us that today is your day to go because your insurance ran out. Then I’ll be like, can I go out the door and come back in, they are like no you can’t.”

Before being incarcerated, several participants developed positive relationships with mental health providers who took Medicaid, whose practices were now located too faraway for participants to be able to comfortably access. “When I first got out, my funds were limited…for me to spend 2 dollars up here and then back seems cheap for the average person but for me it counts,” one participant said. Some participants were released with ankle monitors and one specifically noted that his court-approved provider was outside of the monitor’s programmed bounds: “I didn't have a car back then so I was riding buses. And I had like an ankle monitor for being on parole. And there were times when I would be on the bus going over there [therapy appointment] and this thing would start buzzing.” He described having to call his parole officer to negotiate not receiving a violation while averting his gaze from the bus passengers staring at his monitor.

### Participants express frustration with opaque mental health system

The majority of participants reported being released from prison with limited discharge planning and minimal understanding of what services they would be able to use or where and how to begin. One participant began, “Lot of times I have no idea where to start, you know what I mean, looking for help or asking somebody you know how to do things.” To combat this lack of information on how to access services upon release, almost all participants recommended more support from prison discharge planners. “I want more support to set people up with doctors, mental health, appointments instead of just throwing you out and being like figure it out,” one participant summarized. “Because we don’t have a lot of resources, sometimes people don’t know how to actually go about getting in contact with doctors, making appointments, or getting there…That would be a lot better to make sure we don’t end up back where we just came from. It’s a revolving door.”

Multiple participants shared confusion on which type of provider would be appropriate for their mental health needs. One said, “Um, I think I needed a psychiatrist. Therapist. Um, I don’t really know. Somebody to talk to. Maybe it's some kind of case worker, somebody to help me figure things out.” A second participant was not given information about the mental health services that his primary care facility was able to provide, saying, “I didn’t know that they did, what do you call it, psychiatry. I didn’t know they did that, I thought it was just a primary facility you feel me?”.

Even after being involved in mental health care and receiving services, participants continued to indicate confusion with the types of providers they were engaging with. One participant shared, “I don’t know if it was, what do they call them, I don’t know if it was a case manager or something.” A second attempted to explain, “She was, I think, more or less like a student. She wasn’t a prescriber and she also wasn’t anything like… she was a counselor, but not like a therapist.”

Repeatedly, participants explained that they had multiple types of providers on their care team who were engaged with the participants in varying degrees, and participants report receiving no explanation from the team on each provider’s role. One participant commented, “I talked to this guy for a while, not long. He’s a nice guy. I’m not sure if he was a therapist. [Redacted] was the case manager, so he might have been the therapist?” Another participant was frustrated with not understanding what role her therapist played in her treatment, “I don’t even know what the point is, you know? Like is she [therapist] going to give me advice, is she supposed to point me in the right direction, is she supposed to sit there and just listen to me? I don’t know, I have no idea what she does.”

### Faith and family buffers negative mental health

In the face of obstacles to re-entering society as well as being disappointed by the services they received, multiple participants noted the importance of their faith and family on their mental health. One participant said of his brothers, "They love me even when I was smoking coke, up for three days, looking like shit. I can actually go home and eat. Used to have my brothers come by and pick me up in the car, let's go, you're going home.”

Another shared how her spirituality protected her mental health in the face of distractors, such as substance use, that previously led her away from her goals, “I have to say it’s [faith] 110% my mental health care. I don’t believe what everybody believes but I just believe that something is there and it’s gonna be there. And even if I’m the only one who sees it, I have to maintain discipline and I have to focus.” A third participant said that his family’s belief in God, belief that they passed onto him, intervened in moments of hopelessness and despair: “I've had feelings of giving up. Absolutely. There were days where I just wanted to say ‘f’ it.…but I don't lose hope and faith, I’m a believer. My family, we have a very spiritual background. My mom, my family tells me, 'no matter what, don't give up. Keep pushing. It will get better. If you tell yourself it will get better. And keep striving to make it better. It will happen.”

## Discussion

In this study, we explored the experiences that formerly incarcerated people have while navigating mental health care post-release. Participant responses overwhelmingly emphasized that the principle barriers to accessing and engaging mental health care were housing instability, lack of employment, limited transportation options, and gaps in insurance coverage – all well-known social determinants of health on mental health (Braveman et al., 2011). According to the definition adopted by the Center for Disease Control and Prevention, social determinants of health (SDOH) are the “conditions in the places where people live, learn, work, and play that affect a wide range of health and quality-of life-risks and outcomes” (Centers for Disease Control & Prevention, 2021). By this definition, incarceration, criminal-legal system involvement, and conditions of confinement are clearly SDOH (Massoglia & Pridemore, 2015) and their lasting impacts do not end post-release.

Situating social determinants at the center of mental health, as virtually all of our participants did, challenges a narrow focus on neurobiological causes of mental illness that still persists within psychiatry (Scull, 2021a) and federal research funding (Scull, 2021b). Participants frequently described their negative mental health experiences as produced by the failures of the social safety net and, in a perverse cycle of feedback, these same institutional gaps impeded their access to services that would ostensibly help them. Furthermore, our participants agreed that “structural violence,” such as discrimination from employment due to a criminal record and the subsequent impact that no income has on mental health, cannot be ameliorated or “cured” with therapy and medications—although it often seemed to them that therapists and agencies were offering just those things as responses to their social needs. In reaction to these experiences, participants voiced the need to integrate traditional clinical forms of care with addressing social determinants of health through forms of support that are not limited by the four walls of the clinic.

### Meeting patients where they are

Housing instability was cited by several participants as a key factor contributing to their inability to access and maintain engagement with mental health services. Though only a few were homeless at the time of their interviews, the majority of participants had experienced housing insecurity after being released from incarceration which is substantiated by national data that shows nearly 50,000 people a year enter shelters after release from incarceration (United States Interagency Council on Homelessness, 2016). Moreover, formerly incarcerated people are seven times more likely to be homeless than the general population and these rates are further stratified by race and gender (Couloute, 2018). Although more agencies and health care systems are moving toward a housing first model, few are able to directly supply housing. Still, both professional programs and agencies themselves can equip providers with strategies to better assist clients in unstable environments. As one participant explained, she trusted her caseworker more than her therapist because her caseworker visited her at her tent to provide mental health support in addition to case management services, and connected her to a primary care physician who later provided medical services also at her tent. Currently, many therapists learn that it is impossible to perform in-depth psychotherapy before housing and other foundational needs of Maslow’s hierarchy are met, effectively dissuading them from working with the country’s growing population of long-term and chronically homeless people.

In the context of the type of interlocking systems failures described by our interviewees, suggesting isolated solutions may be inherently misleading since they fail to address the underlying challenges. That being said, there are some immediately available strategies to buffer these structural issues. One example is a street medicine model for behavioral health tailored to people with histories of incarceration. Street medicine was developed to provide health care to individuals experiencing homelessness and requires significant rapport building in non-clinical settings that clients may inhabit, such as community living in shelters or tents. This model of care delivery often pairs health care providers with other services, such as social workers and peer specialists, who are able to buffer the difficulty that participants stated of not having consistent transportation, financial income, or social networks. This public health strategy should not be limited to medical care and can be used for all communities who face institutionalized barriers to accessing services. Importantly, street medicine has already been proven to effectively decrease hospitalization rates, strengthen patient-provider relationships, and maintain ongoing engagement with the service after the initial intervention (Models & in Other Counties: White Paper., 2018). Despite its positive outcomes, street medicine has yet to widely encompass behavioral health care; there are few emerging models such as Los Angeles' first street psychiatry team developed at the Department of Mental Health (Smith, 2022).

Additionally, for several participants, relationships with family and God mitigated negative mental health experiences and inspired hope for making desired changes in one's life. Both social capital (having relationships (Carrasco & Bilal, 2016)) and social cohesion (being in community (Carrasco & Bilal, 2016)) have been identified as important determinants of health that can be modified depending on community (Williams et al., 2020) Participants’ acknowledgement of the importance of familial and religious relationships–relationships developed outside of a clinical setting–provides another strategy for rigorous care. Where appropriate, partnerships between faith organizations and mental health agencies, as well as integrating family and faith communities into post-release planning, are avenues to explore.

### Mental health literacy

Participants reported not having enough information post-release to seek mental health services on their own and once obtained, they had a limited understanding of the roles and services provided. This created confusion and frustration for those who were either unclear about the benefits of or whether they even had access to certain services. These experiences emphasize the need for mental health care providers to promote ‘mental health literacy’. The World Health Organization defines ‘health literacy’ as “the cognitive and social skills which determine the motivation and ability of individuals to gain access to, understand, and use information in ways which promote and maintain good health” (Kanj & Mitic, 2009). Health literacy has since been applied in social and political contexts as a critical tool of empowerment, especially for Black, Indigenous, and other people of color who experience multiple intersections of oppression within health care.

‘Mental health literacy’ involves understanding how to obtain positive mental health and increase help-seeking efficacy (Kutcher et al., 2016). Participants attempted to work with caseworkers, psychiatrists, therapists, and/or counselors without enough information to determine how to work with each role to access usefully individualized care. In this way, mental health literacy operated as an additional social determinant of mental health for participants and its importance cannot be understated in navigating what is generally perceived as a convoluted landscape of mental health agencies, providers, and modalities. To be able to exercise choice, patients must have knowledge of a diverse range of services and how to best utilize them. However, for formerly incarcerated clients such as this study’s participants, it is highly unlikely that the assortment of services they are now presented with on the outside were offered to them within prison walls.

Our participants also reported a lack of systems literacy. Without this ‘literacy’ of the mental health landscape as a whole, it became infeasible to not only self-determine which services would best meet one’s needs but also to interrogate care decisions being made by one’s mental health provider. While prison discharge planning should educate individuals coming home on their options and assist with initial connection to care, there is a broad responsibility, shared by mental health agencies and other levels of the behavioral health system, to better educate potential and current clients on the services offered and how to best utilize them. This education in turn, would strengthen an understanding of what rights individuals have as a patient. One policy recommendation is to expand Medicaid coverage to all incarcerated people thirty days, at minimum, before release. Not only would this reduce some barriers to accessing medications and substance use treatment within the first few weeks of re-entry, it would also give individuals vital time before release to begin the process of figuring out what mental health services are accessible to them within their insurance coverage, an important first step toward building systems literacy.

The problem of mental health literacy also begets a larger, systemic question that future research should investigate: is it possible for a patient to be ‘literate’ within a system that does not itself have an internal consensus on the nature, efficacy, and outcomes of mental health care?

## Conclusion

Participants discussed the importance of shelter, transportation, income, literacy and insurance and, in doing so, pointed out shortcomings in our current model of mental health care in supporting the access and engagement of mental health services by formerly incarcerated people. Because Rhode Island is a small state, with a census population of 1.093 million people, the ‘mental health system’ that participants are navigating is in fact a limited network of providers and agencies. Additionally, because of how small Rhode Island is, there are multiple networks of mutual support that have been developed between formerly incarcerated individuals. This means that this study not only captures a diversity of individual views, but also a broader discussion within a community of formerly incarcerated people. This form of interconnectedness strengthens the importance of understanding how our participants perceive the impact of these determinants on their mental health and their overall understanding of mental health care as it is a community’s response to a specific landscape of care. It is also the discussion that many formerly incarcerated people enter into when first accessing community-based support.

Ultimately, the findings from this study place responsibility on numerous actors, from the legal system to employers to legislators, in addition to mental health professionals, to support and positively influence the mental health outcomes of formerly incarcerated people. One key starting point is recognizing the extraordinary efforts that many formerly incarcerated people make to obtain mental health care that meets their perceived needs. Despite the current efforts being made to address experiences of stigma as well as social determinants of mental health, our participants routinely reported that providers neither understood nor addressed these dimensions of their lives. According to our interviews, this perceived failure of empathy was often more damaging than the failure to successfully address the objective needs.

## Limitations

Limitations sections of qualitative studies often warn that the results are not generalizable. Strictly speaking, transportability is not a form of qualitative validity (Creswell & Poth, 2018). The power of theoretical sampling in a grounded theory-informed approach is that it can develop analysis based on the experiences of a particular, complex community living within specific sociopolitical contexts (Conlon et al., 2020). Following the sociologist Pierre Bourdieu, we see reflexivity as a more rigorous method to explore the limitations of the research process: how does the research process itself participate within the social fields and dynamics that the study is attempting to objectify (Bourdieu & D. Wacquant Loïc J., 2013)?

Participants had been released from incarceration in the last five years. Two of these five years were marked by the COVID-19 pandemic (Sloat, 2022) and a well documented “state of emergency” in Rhode Island's behavioral health system (Declaration of a Rhode Island State of Emergency in Child & Adolescent Mental Health., 2022), including increased demand for services, staffing shortages of 20 to 40 percent, widespread burnout and fatigue among providers, and a mounting housing crisis in the state. Some of the obstacles described in this study may be a combination of long-standing access issues and novel systems failures generated by the combined COVID-19, drug overdose syndemic.

One theme that was not explored in this study, as we became aware of it after interviews had concluded, is how individuals in this population may internalize the lack of a coherent mental health ‘system’ as a personal shortcoming or failure. Further research could explore whether education regarding the current structural challenges and shortcoming of American mental health care might change the ways individuals understand feelings of failure or frustration for not being able to access care and resources.

This study emphasizes participants' perceptions of provider failures over and against exploring how adaptive strategies based on trauma in other contexts, such as incarceration, may have contributed to the way participants both perceived and engaged with services. Given the scope of structural failures in question and the urgency of addressing them–as well as the ways that mental illness can be deployed to blame patients themselves for the absence or shortcomings of social services–we see this potential one-sidedness as appropriate. In future research, however, we will seek to integrate provider perspectives which may complicate both the picture presented here (given that our present focus is on patient perceptions) and add additional dimensions to our understanding of institutional failures, leading to a more comprehensive depiction of dynamics between providers and patients.

## Data Availability

The data from this study is available from the corresponding author on reasonable request. Participant interviews may include details that could lead to identification of participants and this will inform any decision on data requests.

## References

[CR1] American Civil Liberties Union (2014). Briefing Paper: The Dangerous Overuse of Solitary Confinement in the United States.

[CR2] Bourdieu, P., & D. Wacquant Loïc J. (2013). *An invitation to reflexive sociology*. Polity Press.

[CR3] Braveman P, Egerter S, Williams DR (2011). The social determinants of health: Coming of age. Annual Review of Public Health.

[CR4] Bronson J, Berzofsky M (2017). Indicators of Mental Health Problems Reported by Prisoners and Jail Inmates, 2011–12.

[CR5] Carrasco MA, Bilal U (2016). A sign of the times: To have or to be? Social capital or social cohesion?. Social Science & Medicine.

[CR6] Centers for Disease Control and Prevention. (2021). *Social Determinants of Health*. Centers for Disease Control and Prevention. Retrieved from https://www.cdc.gov/socialdeterminants/index.htm

[CR7] Chuang YC, Chuang KY, Yang TH (2013). Social cohesion matters in health. International Journal for Equity in Health.

[CR8] Conlon C, Timonen V, Elliott-O’Dare C, O’Keeffe S, Foley G (2020). Confused About Theoretical Sampling? Engaging Theoretical Sampling in Diverse Grounded Theory Studies. Qualitative Health Research.

[CR9] Couloute, L. (2018). *Nowhere to go: Homelessness among formerly incarcerated people*. Prison Policy Initiative. Retrieved from https://www.prisonpolicy.org/reports/housing.html

[CR10] Creswell, J. W., & Poth, C. N. (2018). *Qualitative Inquiry & Research Design: Choosing among Five approaches*. SAGE.

[CR11] Declaration of a Rhode Island State of Emergency in Child and Adolescent Mental Health. (2022). *Rhode Island medical journal (2013)*, *105*(4), 74. 35476743

[CR12] Evans GW, Wells NM, Moch A (2003). Housing and Mental Health: A Review of the Evidence and a Methodological and Conceptual Critique. Journal of Social Issues.

[CR13] Fellner, J. (2006). A Corrections Quandary: Mental Illness and Prison Rules. Harvard Civil Rights-Civil Liberties Law Review. 41.

[CR14] Frank JW, Wang EA, Nunez-Smith M, Lee H, Comfort M (2014). Discrimination based on criminal record and healthcare utilization among men recently released from prison: A descriptive study. Health & Justice.

[CR15] Kanj M, Mitic W. (2009). Working document: 7th Global Conference on Health Promotion, Promoting Health and Development: closing the implementation gap Nairobi, Kenya, 26–30 October 2009 Geneva (CH): World Health Organization.

[CR16] Knaak S, Mantler E, Szeto A (2017). Mental illness-related stigma in healthcare: Barriers to access and care and evidence-based solutions. Healthcare Management Forum.

[CR17] Kutcher S, Wei Y, Coniglio C (2016). Mental Health Literacy: Past, Present, and Future. Canadian Journal of Psychiatry..

[CR18] Llosa JA, Menéndez-Espina S, Agulló-Tomás E, Rodríguez-Suárez J (2018). Job insecurity and mental health: A meta-analytical review of the consequences of precarious work in clinical disorders. Anales De Psicología.

[CR19] Massoglia M, Pridemore WA (2015). Incarceration and Health. Annual Review of Sociology.

[CR20] Street Medicine Models in Other Counties: White Paper. Bright Research Group. https://www.brightresearchgroup.com/wp-content/uploads/2018/10/Street-Medicine-Models-Summary-of-Findings-9.26.18.pdf. Published 2018.

[CR21] NAMI (2021). Help not handcuffs: Legislation & community models.

[CR22] Pew Center on the States (2011). State of recidivism: The revolving door of America’s prisons.

[CR23] Rotter M, McQuistion HL, Broner N, Steinbacher M (2005). Best practices: The impact of the "incarceration culture" on reentry for adults with mental illness: A training and group treatment model. Psychiatric Services.

[CR24] Sarris J, Logan AC, Akbaraly TN, International Society for Nutritional Psychiatry Research (2015). Nutritional medicine as mainstream in psychiatry. The Lancet. Psychiatry.

[CR25] Scull A (2021). Psychiatry and its Discontents.

[CR26] Scull, A. (2021b). American psychiatry in the new millennium: a critical appraisal. *Psychological medicine*, 1–9. Advance online publication. 10.1017/S0033291721001975 10.1017/S0033291721001975 34158141

[CR27] Simon R, Snow R, Wakeman S (2020). Understanding why patients with substance use disorders leave the hospital against medical advice: A qualitative study. Substance Abuse.

[CR28] Sloat S (2022). *Mental health care should be available for all, not a luxury*. Scientific American.

[CR29] Smith, D. (2022). *L.A'.S first street psychiatrist makes his sidewalk rounds, transforming homeless lives*. Los Angeles Times. Retrieved September 12, 2022, from https://www.latimes.com/california/story/2022-09-07/l-a-countys-first-street-psychiatrist-treats-patients-where-they-live?_amp=true

[CR30] Smith PS (2006). The effects of solitary confinement on prison inmates: A brief history and review of the literature. Crime and Justice.

[CR31] Tong A, Sainsbury P, Craig J (2007). Consolidated criteria for reporting qualitative research (COREQ): A 32-item checklist for interviews and focus groups. International Journal for Quality in Health Care : Journal of the International Society for Quality in Health Care.

[CR32] United States Interagency Council on Homelessness (2016). *Connecting people returning from incarceration with housing and
homelessness assistance*.

[CR33] Vanjani R (2017). On incarceration and health — reframing the discussion. New England Journal of Medicine.

[CR34] Weiss, G., Murphy, A. V., & Salamon, G. (2020). *50 Concepts for a critical phenomenology*. Northwestern University Press.

[CR35] Williams AJ, Maguire K, Morrissey K, Taylor T, Wyatt K (2020). Social cohesion, mental wellbeing and health-related quality of life among a cohort of social housing residents in Cornwall: A Cross Sectional Study. BMC Public Health.

[CR36] Wolff N, Shi J, Siegel JA (2009). Patterns of victimization among male and female inmates: Evidence of an enduring legacy. Violence and Victims.

